# Evaluating the potential of teleneurology in a low‐resource setting for dementia: An exploratory study on patient and healthcare provider satisfaction

**DOI:** 10.1002/alz.094333

**Published:** 2025-01-09

**Authors:** Pullumpallil Thomas Alexander, Mohammad Farhan Ansari, Girish N Rao, Faheem Arshad, Deepak Menon, Priya Treesa Thomas, Subasree Ramakrishnan, Rajani Parthasarathy, Suvarna Alladi

**Affiliations:** ^1^ National Institute of Mental Health and Neurosciences [NIMHANS], Bengaluru, Karnataka India; ^2^ Health and Family Welfare, Government of Karnataka, Bengaluru, Karnataka India

## Abstract

**Background and Objectives:**

Telemedicine, including teleneurology, has emerged as a valuable tool for providing healthcare services remotely, particularly in situations where distance plays a critical role. Teleneurology has the potential to increase access to care, improve patient outcomes, and reduce healthcare costs, especially for patients in rural and underserved areas. We aimed to investigate patients and health care providers satisfaction with teleneurology for dementia.

**Methods:**

We recruited 15 patients with dementia as a part of a public health initiative on Brain Health in the state of Karnataka in India. Patients resided across different districts of the state and had undergone an initial consultation at the specialist centre. Follow up teleconsultations were conducted using the Zoom Video Conference application and included a brief neurological examination. Patient and/or caregiver satisfaction was assessed using Telemedicine Satisfaction Questionnaire (TSQ) and healthcare provider satisfaction was assessed using Patient and Physician Satisfaction with Monitoring Questionnaire (PPSM).

**Results:**

Of the 15 patients with dementia enrolled in the study, mean age of the cohort was 65 years. All 15 patients [8 men] or caregivers completed the Teleneurology Satisfaction Questionnaire (TSQ), yielding a mean score of 4.47±0.41. The mean duration of teleconsultation was 43 minutes. Additionally, the Patient Perception of Satisfaction with Method (PPSM) questionnaire was completed by the neurologist for all 15 patients, resulting in a mean score of 4.33±0.44. Among these patients, 12 consultations were initiated by a primary care physician, and all of them completed the PPSM questionnaire, which had a mean score of 4.04±0.51. Paired patient and neurologist agreement was 60% for quality of care provided, 98% for time saving benefit and 95% for willingness for future use.

**Discussion:**

Overall, patients or their caregivers expressed satisfaction with the quality of care provided through teleneurology and agreed that it was comparable to face‐to‐face encounters. Primary care physicians also reported positive experiences with teleneurology, including a good understanding of patients' health status and the belief that teleneurology was a valuable addition to regular care. Both patients or caregivers and neurologists agreed for the quality of care provided, the time saving benefits and the willingness for future use of teleneurology.

